# The Lunchbox Study: A Pilot Examination of Packed Lunches of Children with Autism Spectrum Disorder

**DOI:** 10.3390/nu14071338

**Published:** 2022-03-23

**Authors:** Laura Seiverling, Jennifer Felber, Monica Howard, Keith Williams, Helen M. Hendy

**Affiliations:** 1 Special Education Department, Ball State University, 2000 W University Avenue, Muncie, IN 47306, USA; 2 The Summit Center, 150 Stahl Road, Getzville, NY 14068, USA; jfelber@thesummitcenter.org; 3 The ELIJA School, 11 Laurel Lane, Levittown, NY 11756, USA; mhoward@elijaschool.org; 4 Penn State Hershey Medical Center, 905 West Governor Road, Hershey, PA 17033, USA; kwilliams2@pennstatehealth.psu.edu; 5 Psychology Program, Schuylkill Campus, Penn State University, 200 University Drive, Schuylkill Haven, PA 17972, USA; hl4@psu.edu

**Keywords:** autism spectrum disorder, packed lunches, fruit and vegetable consumption

## Abstract

Background: This study examined foods packed and consumed by children with autism spectrum disorder (ASD) and calculated the percentage of packed school lunches meeting National School Lunch Program (NSLP) standards. Fruit and vegetable (FV) consumption was further examined by investigating its association with the number and type of foods packed. Methods: Participants included 59 private school students observed for five school meals. Servings of foods and beverages packed and consumed and the percentages of correspondence between food packed and consumed were calculated. Next, the percentages of lunches meeting NSLP guidelines were calculated. Finally, mealtime means were calculated for the number of foods packed, FVs packed, and FVs consumed. Results: There was a high correspondence between foods packed and consumed. Fresh fruits and sugar-added drinks were most often packed and consumed. The percentage of meals meeting NSLP guidelines was higher than previous non-ASD samples. More FV consumption was associated with more FVs packed and fewer total foods packed. FV consumption was highest with 4 < 5 foods packed, including 2+ FVs. Conclusions: Future studies should compare foods packed and consumed by children with and without ASD. The FV consumption findings suggest parents may increase children’s FV consumption by packing approximately four total foods with two FVs.

## 1. Introduction

Several studies have examined caregiver-provided packed lunches and found both a low frequency of fruits and vegetables and a high frequency of energy-dense discretionary foods in these lunches. For example, one study examined the packed lunches of 607 preschoolers on two nonconsecutive days and found that sugar exceeded the daily recommended intake in 29% of lunches and that these lunches were commonly low in fiber and nutrients, such as potassium [1]. This study also found that sugar-sweetened beverages were more commonly packed than other beverages. Another study examined 927 lunchboxes and found that not only were foods high in fat, sodium and sugar over-represented, but children often did not consume healthy foods, such as fruits and vegetables [2]. Hubbard et al. [3] examined the packed snacks and lunches of 626 elementary school children to determine the frequency in which these lunches contained foods meeting the National School Lunch Program Standards (NSLP) [4] and found three or more standards were met by only 27% of packed lunches. 

To date, packed lunches of children with autism spectrum disorder (ASD) have not been an object of study, despite a growing literature showing that children with ASD have more limited diets than children without ASD [5,6,7,8]. While some studies have examined only the contents of the packed lunches [3], others have examined both the foods packed and the foods consumed [2]. A main goal of the current study was to provide an initial examination of the packed lunches of children with ASD by reporting the average servings of beverages and foods packed and consumed across five school lunches. Next, the percentage of packed school lunches meeting each of the NSLP standards was compared to the findings to those of Hubbard et al.’s study [3] with a large sample of general elementary students. We hypothesized the nutritional quality of foods consumed from packed lunches would be lower for our sample of students with ASD compared to Hubbard’s previous findings involving a more diverse student sample. Further, because children with ASD tend to have more limited and less healthy diets than those without ASD, we specifically examined the relationship between the number of foods packed and fruit and vegetable consumption. 

## 2. Methods

### 2.1. Participants

Study participants included 59 students and their parents (mean age = 13.8 years; 78.0% males) from five private schools specializing in the education of children with ASD. The research was conducted according to the guidelines of the Declaration of Helsinki, and approved by the Penn State College of Medicine Institutional Review Board. As our goal was to examine a range of children with ASD, children were not selected based on their eating habits or nutritional status. Two schools were in New York, and one each in Connecticut, Pennsylvania and New Jersey, all of which allow children to bring packed school lunches. In three of the schools, all students brought packed lunches, while two schools offered school-provided lunches as well. (Table 1 shows descriptive statistics of the student demographics and study variables.) 

### 2.2. Procedures

Parents were given brief surveys that asked them to report their child’s demographic characteristics, including age, gender, height and weight, which were used to calculate body mass index z-scores (BMIz) for those students under 18 years of age. The surveys also asked parents to report whether their child would eat at least one tablespoon of 15 common fruits and 15 common vegetables. (Table 2 shows a list of these foods and the percentages of students who accepted them, as reported by parents). 

Then, the packed lunches of the 59 students were observed for five meals, each across two to three weeks to record the number and types of beverages and foods offered; the number of these items for which *any amount was consumed*, as well as the number of FVs included in the lunch, and the number of FVs for which *at least 50% of the amount was consumed.* Trained school staff made these lunchtime observations, all using the same datasheet and definitions of study variables. For each lunch observation, staff recorded each food and drink contained in the child’s lunch, including a description of the item, such as with the brand and volume (e.g., peanut butter and jelly sandwich, six-ounce Juicy Juice ™ apple juice pouch). At the end of the meal, the staff recorded the amount consumed. To encourage accuracy, the datasheets included samples of food item descriptions, as well as examples of pre- and post-lunch measures. 

For the FV observations, inter-rater reliability data were collected for 27% (i.e., 23 out of 80) lunches at two of the five schools by having a second staff member independently record lunch observations. Inter-rater reliability was calculated for each of the four measures: type of food packed, amount of food packed, post-lunch amount consumed. If both observers recorded the same data for a food, this was scored as an agreement. If the data were different, this was scored as a non-agreement. For each meal, we calculated the percentage of agreement for each measure by dividing the total number of packed lunch items by the number of agreements. These percentages were then averaged across the 23 meals. The inter-rater agreement was 100% for the type and amount of food packed. For the food amount consumed, the inter-rater agreement was 95% (75%–100%). 

### 2.3. Dietary Coding

Hubbard and her colleagues [3] used 26 different categories of food and drink to classify the contents of packed lunches. As each of the categories included a clear description and the categories could describe a wide range of foods, such as pizza or ethnic dishes, we used their food classification system in this study. Using the meal datasheets, one of the primary investigators categorized every food or drink in the packed lunch into one of the 26 food categories utilized by Hubbard et al. [3]. This was done for all five lunch observations for all children in the study. If the datasheet indicated that *any portion of a food was consumed*, the food was scored as both packed and consumed. If the datasheet indicated that a food was packed and none was consumed, it was scored only as food packed. To examine the relationship between the foods packed and foods consumed, a percentage of correspondence was obtained by dividing the food packed by the food consumed for each of the 26 food categories (Table 3 shows the average number of servings of beverages and foods packed and consumed across five school lunches, as well as the percentage of correspondence between beverages and foods packed and consumed.).

### 2.4. Evaluation of Lunch Quality

To evaluate the quality of the lunches, we evaluated each of the 295 lunches (59 participants × 5 lunch observations) using the five standards of the National School Lunch Program (NSLP) [9]. We examined both the percentage of lunches meeting each standard and the number of standards met per lunch. We also compared our results to the data of packed lunches by students without ASD presented by Hubbard and her colleagues [3]. 

The five NSLP standards are as follows:

1. 1/2 cup of fruit (excludes fruit juice);
2. 3/4 cup of vegetables (excludes vegetable juice and vegetables “carried” in another item, such as lettuce on a sandwich because the contribution to total vegetable portion size was negligible);
3. One ounce of grains from bread, rice, pasta, cereal and granola (excludes grains from snack foods and desserts);
4. One ounce of meat/meat alternate (from sandwiches with protein filling, nuts/seeds, eggs, peanut/nut butter, hummus, leftover meat, cheese and yogurt);
5. One cup of fluid milk.

While Hubbard and her colleagues examined only whether an appropriate portion of a food meeting an NSLP standard was included in the packed lunch, we examined whether a portion of food required to meet the standard was consumed (Table 4 shows a comparison of NSLP standards met by the lunch foods observed in the present sample of students with ASD and the previous Hubbard sample of students with typical development.).

### 2.5. Evaluation of FV Consumption 

Because children with ASD tend to have more limited diets compared to those without ASD [5,6,7,8], we further examined how foods packed for lunch were associated with FV consumption in the current sample. Instead of counting any portion of the FVs as consumed for this evaluation as done with the food categories listed in Table 2, we used the lunch datasheets to determine across the five observed meals the *mean number of FVs consumed* of at least 50% of the amount packed to ensure that at least half of the portion of FVs packed were eaten by the student in order to count each FV as consumed. Additionally, the datasheets were used to determine across the five meals the *mean number of all foods packed* in the lunches, and the *mean number of FVs packed* in the lunches. 

## 3. Results

### 3.1. Descriptive Statistics

Table 1 shows descriptive statistics for student demographics and study variables relevant to the examination of FV consumption. Table 2 shows the percentage of students whose parents reported they would consume at least one tablespoon of 15 common fruits and 15 common vegetables. The seven most accepted fruits were apples, bananas, grapes, watermelon, strawberries, oranges and pears. The seven most accepted vegetables were potatoes, corn, carrots, cucumbers, green beans, lettuce and sweet potatoes. 

Table 3 shows the servings of beverages and foods packed and consumed (*in any amount*), as well as the percentage of correspondence between items packed and consumed. We found a high correspondence between foods packed and at least a portion of the foods consumed, with a range of 80–100% and an average of 94.7%. Fresh fruit was found to be the food category most often packed and consumed. Sugar-added drinks were the beverages most often packed and consumed, while milk and calorie-free beverages were rarely packed or consumed. In this sample, leftovers (either a grain dish or a meat/protein dish) were packed more than sandwiches, which differs from the findings of Hubbard and colleagues [3] who found that students packed sandwiches more than three times as often as leftovers. 

Table 4 shows the percentage of packed school lunches meeting each of the NSLP standards in the current study and the Hubbard et al. study [3] in a sample of children without ASD and the total number of lunch standards met. The percentage of meals in this sample, which met NSLP standards, was higher than the percentages found by the Hubbard et al. study [3] for each of the standards except milk. While children in the current sample never met the standard for milk, they met the standards for both grain and protein for most lunches.

### 3.2. Student Demographics and FV Consumption

A preliminary goal for data analysis of the present study was an examination of whether student demographics (school, gender, age, BMIz) were associated with mean FV consumption across five observed meals. Using SPSS 27 software, a one-way ANOVA was first used to compare the five schools for mean FV consumption, finding no significant effects (*F*_(4,54)_ = 1.78, *p* < 0.148). Then, ANCOVA was used to examine gender, age and BMIz for associations with the mean FV consumption, again finding no significant effects for these demographics (*F*_(1,44)_ = 2.54, *p* < 0.120; *F*_(1,44)_ = 0.21, *p* < 0.654; *F*_(1,44)_ = 1.84, *p* < 0.183; respectively).

### 3.3. Number and Type of Foods Packed and Student FV Consumption

The primary goal for data analysis was an examination of both the *number of foods packed* for lunch and the *number of FVs packed* for lunch for their association with *mean FV consumption*. Because it considers each predictor for its association with the outcome variable with other predictors already taken into account, multiple regression analysis was used. (See Table 5.) Results of the multiple regression analysis revealed that more FV consumption was seen for the students with ASD when more FVs were packed in their lunches (*p* < 0.001) and when fewer total foods were included (*p* < 0.026).

**Figure 1:**
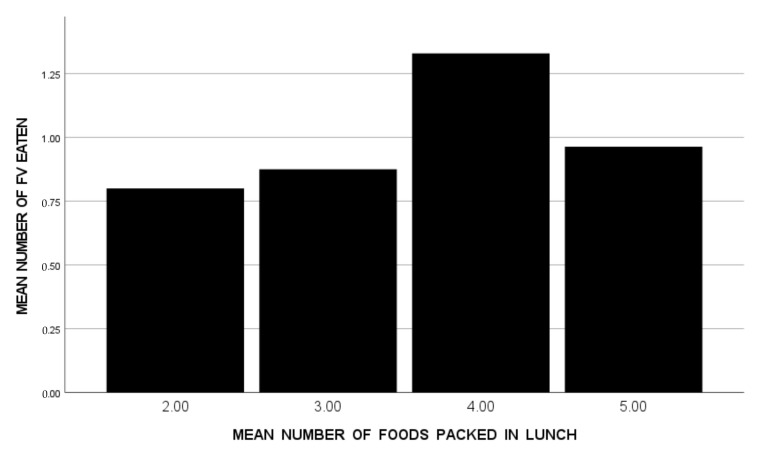
Mean Number of FV Eaten Across Five School Lunches by Students with ASD (*n* = 59), Divided into Groups According to the Mean Number of Foods Packed in Their Lunches (*n* = 15 for <3, *n* = 16 for 3 < 4, *n* = 17 for 4 < 5, *n* = 11 for 5+).

**Figure 2:**
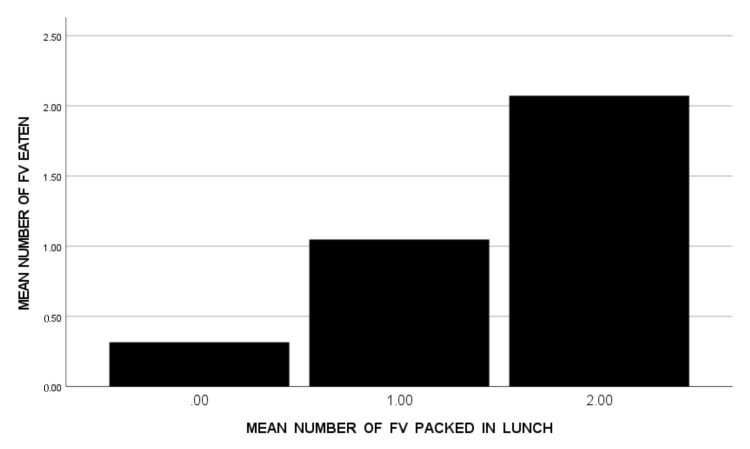
Mean Number of FV Eaten Across Five School Lunches by Students with ASD (*n* = 59), Divided into Groups According to the Mean Number of FV Packed in Their Lunches (*n* = 19 for <1, *n* = 29 for 1 < 2, *n* = 11 for 2+).

Then, to identify the specific numbers of total foods and FVs packed that were optimally associated with FV consumption, the 59 students with ASD were clustered into groups according to their mean number of total foods packed (<3, 3 < 4, 4 < 5, 5+), and then into groups according to their mean number of FVs packed (<1, 1 < 2, 2+). Bar graphs were used to show mean FV consumption for each of these groups. (See Figure 1 and Figure 2) A visual scan of these graphs suggests that the highest FV consumption was shown by the students with ASD when their lunches included 4 < 5 total foods (Figure 1) with 2+ FV foods (Figure 2).

## 4. Discussion

These findings expand upon the current school lunch literature by examining the nutritional quality of packed lunches among a sample of students with ASD. We found a high correspondence between foods packed and at least a portion of those foods consumed, suggesting that parents primarily offer foods they know their children will eat. This is not a surprising finding, given past research has found that not only do caregivers tend to offer foods to their children that they know their children will eat [10], but the dietary patterns of children with ASD can even affect what other members of the family eat [6].

Despite the fact that we examined whether the child actually ate the food required to meet the NSLP standards, and Hubbard and colleagues [3] only examined whether the foods were included in the packed lunches, the percentage of meals meeting four of five NSLP standards was higher for the current sample of children with ASD. This finding was unexpected and seems to be inconsistent with previous studies, which showed children with ASD eat fewer healthy foods than their same-aged peers without ASD [5,7]. We believe the children with ASD in this sample may have had access to a wider range of foods, which allowed them to more easily meet the NSLP standards. The frequency of foods from Hubbard and colleagues’ [3] broad category of “leftovers”, which included mixed dishes, such as ethnic foods, pizza-like foods—pizza or pizza-based hot pockets—or grains such as rice were found more often in the lunches of the children with ASD than the schoolchildren in Hubbard’s study. In all the private schools that participated in our study, school staff would warm foods in a microwave and help prepare foods, as required, which may have increased the likelihood that parents would pack foods that required heating. It is not known if the public schools participating in Hubbard’s study either had access to a microwave or if school staff could warm or prepare food, but we suspect they did not. It is possible the parents of children who attend public schools pack their children’s lunches without the expectation of having food heated or prepared, thus limiting the foods included in packed lunches. While it may be possible to send warm foods in an insulated container, this may not be feasible for many families. The availability of having food warmed could be a factor leading to increased dietary quality of packed lunches. While not advocating that all schools must have the capacity to warm foods in packed lunches, further research should examine whether dietary quality varies based upon the availability of heating foods from packed lunches.

Another surprising finding was that none of the students in our sample met the national standard for milk consumption. While we believe this is largely due to parental reluctance to send a beverage that requires refrigeration, other factors include diet limitations or family selection of a gluten-casein free diet for their child with ASD. This finding should also be explored in future research by examining milk consumption in both home and school settings in the ASD population.

Although, overall, there was a strong correspondence between foods packed and foods consumed in the lunches packed and analyzed for this study, the closer examination of the *number of foods packed* for lunch and the *number of FVs packed* for lunch for their association with *mean FV consumption* suggests that caregivers who may be interested in having their children consume more FVs during school meals may benefit from guidance on which, and how many foods to include in their children’s packed lunches. The simple suggestion of initially selecting a fruit and/or vegetable (perhaps chosen from those most accepted) and then selecting only two or three additional foods may increase the probability that the child will consume the fruit or vegetable. Even though interventions to improve diet quality of school lunches have generally been shown to have a positive impact, especially on the presentation of vegetables [11,12,13], these interventions, to date, have been far more complicated than limiting the number of foods included in the packed lunch. If a parent has been supplying five or more foods in the packed lunch, an intervention simply limiting lunch to a fruit or vegetable and two or three additional foods would decrease the parent’s response effort, perhaps improving both the acceptability and maintenance of the intervention.

Our observation that five or more foods in a packed lunch are related to lower consumption of fruit and vegetables than lunches with fewer foods did not occur in the context of an intervention, but rather a study on the diet quality of the packed lunches of children with ASD without known eating difficulties; however, we believe this finding might be generalizable to a wider population of children and could be used in the development of a simple, low-cost intervention for increasing fruit and vegetable consumption. Future research could involve interventions based upon limiting the number of foods included in packed lunches along with guidance on food choice (i.e., include a fruit or vegetable). Most studies of packed lunches typically focus on which foods are included in the lunch and not on which foods are consumed [3]. Although there is often a strong correlation between foods packed and consumed, what is packed is not always consumed. We suggest that future intervention studies include food consumption as an outcome measure.

Although we gathered no information involving the rationale parents used for including specific foods in their children’s lunches, we speculate parents who were concerned their children would not get enough to eat included more foods in their children’s lunches. While including more foods in the lunch may have increased calorie intake, it may also result in a decreased fruit and vegetable intake. Providing parents with the information that simply adding more foods to the packed lunch may not improve the nutritional value of the lunch, especially if the additional foods are discretionary foods, may influence the type and number of foods parents include in the packed lunches.

There are a number of limitations to the current study worth noting that should be addressed by future researchers. First, it is unclear how generalizable these findings are, as the sample included five private schools serving children with ASD. Future researchers should examine if similar findings are obtained when examining packed lunches of students with ASD in public school settings. Further, although the current findings were compared to those of Hubbard et al. [3] with a sample of elementary school students, a comparison group of non-ASD students was not included in the current study and the methods used in the current study (i.e., measuring consumption across five meals) and the Hubbard et al. study (i.e., measuring foods packed in one lunch) differed. Future studies should compare the nutritional quality of packed lunches in children with and without ASD by using consistent measures for tracking foods packed and consumed.

Additional limitations of the project include the lack of inter-rater reliability for all school locations, as inter-rater reliability was only collected for two locations. Further, when examining FV consumption, the definition for consumption was at least 50% of the portion packed, and the definition of consumption was *any* of the portion consumed for the food categories listed in Table 2. Although on most lunch datasheets, students who consumed any portion almost always consumed at least 50% of the portion, future researchers should use consistent definitions for consumption and consider various methods (e.g., taking photos of foods or weighing foods before and after meals) to most accurately measure the intake during meals.

## 5. Conclusions

To our knowledge, this was the first study to examine the nutritional quality of packed school lunches for students with ASD. Contrary to our hypothesis, the percentage of school lunches packed exceeded each of the NSLP standards compared to earlier studies with non-ASD samples, and while FV consumption was found to be highest during meals when more FVs were packed and when fewer total foods were included, it is unclear if these findings are unique to the current sample of students with ASD attending a private school. Future research should further examine and compare lunch mealtime consumption (of both packed and school-provided lunches) of students with and without ASD in order to investigate if these preliminary findings generalize to larger and more diverse samples of students.

## Figures and Tables

**Table 1:** Descriptive Statistics for Demographics and Study Variables for Students with ASD (*n* = 59).

Variable	%		
School location			
1	15.3%		
2	11.9%		
3	23.7%		
4	18.6%		
5	30.5%		
Male gender	78.0%		
Variable	*M*	*SD*	*Range*
Student age (years)	13.80	5.09	5.00 to 20.00
BMI z-score	0.46	1.46	–3.45 to 2.98
Mean number foods packed	3.75	1.19	2.00 to 8.20
Mean number FV packed	1.22	0.87	0.00 to 4.60
Mean number FV consumed	1.00	0.77	0.00 to 2.80

**Table 2:** Percentages of Students with ASD Reported by Parents to Eat at Least One Tablespoon of Each Common Fruit or Vegetable (*n* = 54). Also Shown are Seven Most Accepted Fruits and Vegetables.

Fruits	%	Vegetables	%
Bananas	77.8%	Potatoes	66.7%
Apples	83.3%	Tomatoes	44.4%
Grapes	74.1%	Onions	29.6%
Strawberries	72.2%	Carrots	64.8%
Oranges	70.4%	Lettuce	50.0%
Watermelon	74.1%	Peppers	46.3%
Blueberries	59.3%	Celery	35.2%
Peaches	61.1%	Cucumbers	59.3%
Pineapple	59.3%	Corn	64.8%
Cherries	37.0%	Sweet potatoes	50.0%
Pears	70.4%	Green beans	53.7%
Raisins	57.4%	Cauliflower	42.6%
Plums	46.3%	Peas	46.3%
Nectarines	61.1%	Squash	33.3%
Grapefruit	22.2%	Lima beans	24.1%
Top Seven Fruits:	%	Top Seven Vegetables:	%
Apples	83.3%	Potatoes	66.7%
Bananas	77.8%	Carrots	64.8%
Grapes	74.1%	Corn	64.8%
Watermelon	74.1%	Cucumbers	59.3%
Strawberries	72.2%	Green beans	53.7%
Oranges	70.4%	Lettuce	50.0%
Pears	70.4%	Sweet potatoes	50.0%

**Table 3:** Mean Number of Servings of Beverages and Foods Packed and Consumed (In Any Amount) Across Five School Lunches by Students with ASD (*n* = 59). Also Shown is the Percentage of Foods Packed that are Consumed.

Categories	Mean Foods Packed	Mean Foods Consumed	% (Consumed/Packed)
Beverages			
Water	1.03	0.83	84%
Sugar-sweetened	0.92	0.91	986%
Milk	0.14	0.12	83%
Juice	0.60	0.58	100%
Calorie-free	0.03	0.03	100%
Sandwiches			
Protein filling	1.03	1.00	93%
Fat or carb filling	1.02	1.00	98%
Snack foods			
Chips, pretzels	1.28	1.22	98%
Crackers	0.78	0.74	99%
Nuts, seeds	0.12	0.12	100%
Fruits			
Fresh	2.45	2.31	95%
Canned	0.58	0.51	80%
Dried	0.09	0.09	100%
Desserts			
Cookies	1.11	1.08	98%
Baked goods	0.74	0.71	98%
Other desserts	0.34	0.28	85%
Leftovers			
Grains	1.23	1.17	96%
Meat, protein	1.38	1.37	99%
Mixed dish	0.43	0.42	93%
Pizza	0.49	0.49	100%
Dairy foods			
Yogurt	0.88	0.83	96%
Cheese	0.28	0.25	91%
Vegetables			
Green, orange, red	0.83	0.74	95%
Starchy, other	0.29	0.29	100%

**Table 4:** Percentages of Packed School Lunches Meeting the NSLP Guidelines for the Present Sample of Students with ASD with 295 Lunches Observed, and for a Previous Study of Children with Typical Development by Hubbard et al. (2014) with 626 Lunches Observed. Also Shown are the Total Number of NSLP Guidelines Met.

National School Lunch Guideline	Present Study: % of Packed Lunches Meeting the Guideline	Previous Study: % of Packed Lunches Meeting the Guideline
½ cup fruit	38%	32%
¾ cup vegetable	14%	6%
1 oz. grains	67%	65%
1 oz. protein	77%	66%
8 oz. milk	0%	15%
Total Number of Lunch Guidelines Met:		
0	5%	20%
1	21%	38%
2	50%	38%
3	24%	23%
4	1%	5%
5	0%	0%
Met 3 or more guidelines	25%	27%

**Table 5:** Multiple Regression to Examine Mean Total Number of Foods Packed and Mean Number of FV Packed as Predictors of Mean FV Consumed Across Five School Lunches by Students with ASD (*n* = 59).

Variable	*Beta*	*t*	*p*
Mean Number of Foods Packed	–0.177	2.30	0.027
Mean Number FV Packed	0.765	9.92	0.001
	*R^2^* = 0.695		
	*F*_(2,56)_ = 63.94		
	*p* < 0.001		

## Data Availability

The data presented in this study are available on request from the corresponding author. The data are not publicly available due to participant demographic data included (i.e., date of birth, height and weight).
